# RESILIENCE AND HEALTH AMONG CHINESE OLDER ADULTS: FINDINGS FROM THE U.S. AND CHINA

**DOI:** 10.1093/geroni/igz038.2186

**Published:** 2019-11-08

**Authors:** Wei Zhang, Bei Wu, David Matchar

**Affiliations:** 1 University of Hawaii at Manoa, Honolulu, Hawaii, United States; 2 New York University, New York, New York, United States; 3 Duke-NUS Medical School, Singapore, Singapore, Singapore

## Abstract

The new framework of resilient aging has gained its importance in recent years. This symposium provides new findings on resilience and health among the Chinese population. Using data collected among 430 Chinese older adults in Honolulu, the first presentation examines resilience as an explanatory mechanism linking neighborhood social environment and well-being. Results showed that neighborhood cohesion was positively related to psychological well-being and life satisfaction. Resilience contributed to a substantial portion of the associations. Using the same data, the second presentation examines the association between immigrant status and oral health related quality of life (OHQoL) and the moderating role of resilience. Findings showed that U.S.-born Chinese immigrant older adults had better OHQoL than their foreign-born Chinese American counterparts. Resilience was positively associated with OHQoL for the former but not for the latter. The third paper presents findings from the same dataset along with a survey of 800 older adults in Wuhan, China. The positive relationship between attitudes towards aging and self-rated health (SRH) was found to be moderated by resilience such that higher levels of resilience weakened this association substantially. Both the positive focal relationship and the moderating effect appeared to be stronger among participants in Honolulu. Using both datasets, the fourth paper investigates patterns of intergenerational transfer and their relationships with SRH as well as the meditating effect of resilience. Findings highlighted the beneficial health effects of receiving emotional support from adult children as well as the mediating role of resilience for older females in both study sites.

